# Complete genome sequence of multidrug-resistant *Salmonella enterica* subsp. *enterica* serovar Enteritidis SD191 isolated from chicken liver, harboring a novel imipenem resistance mechanism

**DOI:** 10.1128/mra.00239-26

**Published:** 2026-09-08

**Authors:** Chenyi Xie, Xin Yu, Xiange Dong, Yuxin Yang, Yang Liu, Jianlong Zhang, Jiayu Yu, Jiuyu Zhan, Hongwei Zhu, Xingxiao Zhang

**Affiliations:** 1 School of Life Sciences, Ludong University 686533, Yantai, China; 2 Yantai Key Laboratory of Animal Pathogenetic Microbiology and Immunology, Yantai, China; University of Pittsburgh School of Medicine, Pittsburgh, Pennsylvania, USA

**Keywords:** antibiotic resistance

## Abstract

We present the complete genome sequence of *Salmonella enteric*a subsp. *enterica* serovar Enteritidis SD191 isolated from *Gallus gallus* liver in China, harboring plasmid pSE191. The genome reveals multiple antibiotic resistance mechanisms and phenotypic imipenem resistance without canonical genes.

## ANNOUNCEMENT

Antimicrobial resistance in bacterial populations from food-producing animals poses a significant public health concern (1). Carbapenems are classified as “critically important” antimicrobials and remain one of the final therapeutic options for severe infections caused by multidrug-resistant bacteria (2). Here, we sequenced the genome of SD191 isolated from a *Gallus gallus* farm in Shandong, China (34.89° N, 118.11° E) to investigate its genetic determinants of enhanced virulence and resistance.

Five liver samples were aseptically collected from *G. gallus* carcasses exhibiting symptoms of Salmonellosis and delivered to the Key Laboratory of Animal Pathogenetic Microbiology and Immunology for pathogen detection. Approximately 1 g of tissue was homogenized in 9 mL of sterile saline and enriched in selenite cystine broth (SCB) at 37°C for 24 h. The enriched cultures were streaked onto Xylose Lysine Tergitol 4 (XLT4) agar and incubated for 24 h at 37°C under aerobic conditions. Black colonies indicative of *Salmonella* spp. were purified by passaging three times. The purity of the culture was confirmed by uniform colony morphology. Antigenic formulas were determined by *Salmonella* agglutinating antisera pools using standard methods (ISO/TR 6579-3:2014), assigning the strain as serovar Enteritidis per the White–Kauffmann–Le Minor scheme (1).

Genomic DNA was extracted using the DNeasy Blood & Tissue Kit (Qiagen, Germany). Two libraries were prepared: a short-read library using the NEBNext Ultra II DNA Library Prep Kit for Illumina, and a long-read library using the SMRTbell Express Template Prep Kit 2.0 for PacBio (all bioinformatic tools were employed with default parameters unless otherwise specified). Sequencing was performed on the Illumina NovaSeq 6000 (paired-end 2 × 150 bp) and PacBio Sequel II platforms. A total of 4,651,358 reads were generated. Quality control was performed using FastQC v0.11.9, and low-quality reads were filtered using Trimmomatic v0.39. Hybrid assembly was performed using Unicycler v0.4.8, resulting in a circularized chromosome and one plasmid. Polishing was conducted using Pilon v1.23. The origin of replication was determined using DoriC 10.0. The genome of SD191 consists of a circular chromosome of 4,677,442 bp (guanine–cytosine [GC] content: 52.1%) and one plasmid, pSE191, of 24,484 bp (GC content: 44.5%). The assembly coverage was 50× for the chromosome and 50× for the plasmid. The annotation predicted 4,301 coding sequences (CDSs).

The plasmid sequence was identified using PlasFlow v1.1. Annotation was performed using the National Center for Biotechnology Information (NCBI) Prokaryotic Genome Annotation Pipeline (PGAP) (3). Antimicrobial resistance genes were identified using the Comprehensive Antibiotic Resistance Database (CARD) via the RGI tool (v5.2.0) with “Perfect” and “Strict parameters (4).

Phenotypic susceptibility testing revealed that SD191 is a multidrug-resistant (MDR) strain. Minimum inhibitory concentrations (MICs) were determined by broth microdilution according to Clinical and Laboratory Standards Institute (CLSI) guidelines . The strain exhibited resistance to imipenem (MIC > 4 µg/mL). The RGI analysis identified 28 antibiotic resistance ontology (ARO) terms, including genes conferring resistance to aminoglycosides (*APH(6)-Id* ARO:3002660), beta-lactams (*TEM-1* ARO:3000873), and fluoroquinolones (*gyrA* mutations). Notably, no canonical carbapenemase genes (e.g., *blaKPC* and *blaNDM*) were detected, suggesting a novel mechanism of imipenem resistance in SD191 potentially linked to efflux pump overexpression or porin loss.

## Data Availability

The complete genome sequence of *Salmonella enterica* subsp. *enterica* serovar Enteritidis SD191 has been deposited in DDBJ/ENA/GenBank under accession CP196754 (chromosome) and CP196755 (pSE191), and the Illumina raw data accession number is SRR35737863, and the PacBio raw data accession number is PRJNA1289005.
